# The Mental Health of Poles during the COVID-19 Pandemic

**DOI:** 10.3390/ijerph20032000

**Published:** 2023-01-21

**Authors:** Karolina Hoffmann, Dorota Kopciuch, Aleksandra Bońka, Michał Michalak, Wiesław Bryl, Krzysztof Kus, Elżbieta Nowakowska, Tomasz Zaprutko, Piotr Ratajczak, Anna Paczkowska

**Affiliations:** 1 Department of Internal Diseases, Metabolic Disorders and Arterial Hypertension, Poznan University of Medical Sciences, 60-572 Poznań, Poland; 2 Department of Pharmacoeconomics and Social Pharmacy, Poznan University of Medical Sciences, 60-806 Poznań, Poland; 3 Department of Computer Science and Statistics, Poznan University of Medical Sciences, 60-806 Poznań, Poland; 4 Department of Pharmacology and Toxicology, Institute of Health Sciences, Collegium Medicum, University of Zielona Góra, 65-516 Zielona Góra, Poland

**Keywords:** COVID-19, mental health, depression, anxiety, stress, insomnia

## Abstract

Background: The SARS-Cov-2 pandemic has had a profound impact on all aspects of life in the global population, causing above other, psychological problems. Aims: The objective of this study was to evaluate the mental health of the Poles during the COVID-19 pandemic. Methods: A prospective, cross-sectional web-based survey design was adopted. The study horizon was from 1 June 2021, to 31 December 2021. An anonymous, standardized questionnaire was disseminated electronically by means of social media among Polish adults. The following tests were performed: Depression, Anxiety, and Stress Scale (DASS-21), and the Insomnia Severity Index (ISI). Results: A total of 1306 individuals completed the survey. Of the participants, 77.79% were female at a mean age of 34.89 ± (14.79), 62.25% had higher education, and 56.43% were employed. The prevalence of depression, anxiety, stress, and sleep disturbances in this sample were as follows: 50.38%, 43.49%, 61.26%, and 44.74%, respectively. Poor self-estimated health status, the presence of comorbidities, and regular use of nicotine significantly increased the risk of any of the analyzed psychiatric symptoms and sleep disorders. The depression level was significantly associated with age, living alone, health status, and the use of nicotine. Moreover, the level of anxiety was significantly associated with age and health status. The level of stress depended on gender, age, health status, use of nicotine, and being vaccinated against SARS-CoV-2. Sleep disturbances depended on age, health status, the presence of comorbidities, and regular use of drugs. Conclusions: The Polish population manifested numerous psychological symptoms during the COVID-19 pandemic. There is a need to afford psychological support to them and ensure their mental health.

## 1. Introduction

Almost three years have passed since World Health Organization (WHO) declared the coronavirus disease 19 (COVID-19) crisis a pandemic [1,2]. COVID-19 is caused by a virus originally named ‘2019 novel coronavirus’ (2019-nCov), and then, in February 2020, it was renamed ‘severe acute respiratory syndrome coronavirus 2′ (SARS-CoV-2) [1]. The first case of COVID-19 was detected in 2019 in Wuhan City of Hubei province [1,2]. The worldwide spread of the disease soon followed, with serious consequences due to its unpredictable course, complex pathogenesis, and lack of effective treatment. In Poland, the first case of COVID-19 was detected on 4 March 2020 [3]. Nowadays, Poland is ranked the 21st country in terms of the number of infections and the 15th in the number of deaths in the world [4]. On 16 January 2023, there were 671,464,425 confirmed cases of COVID-19 worldwide, and death occurred in 6,731,268 patients. In Poland, there were 6,373,370 total cases and 118,651 total deaths [4].

In Poland, preventive sanitary measures were promptly implemented. In the period 2020–2022 numerous restrictions based on the relevant regulations of the Council of Ministers were introduced and updated according to changing sanitary situation [5]. In the period from 14 to 20 March 2020, a state of epidemic threat was in force in Poland [6], and from 15 March 2020, a sanitary cordon was introduced at the borders of Poland, significantly limiting border traffic [7,8]. From 20 March 2020 to 15 May 2022, in accordance with the regulation of the Minister of Health, the state of epidemic was in force in Poland [9]. From 16 May 2022, the state of epidemic threat is in force again [10].

From 1 June 2021 to 31 December 2021, sanitary restrictions were not as strict as during the first three waves of the COVID-19 pandemic in Poland. The number of total cases increased from 2,872,868 to 4,108,215, and the number of total deaths from 73,856 to 97,054 [4]. On 19 April 2021, kindergartens and nurseries were allowed to open, and sports competitions in groups of up to 25 people in the open were allowed. In May 2021, due to the decrease in the number of infections, most restrictions were lifted (cultural places, stadiums, schools, etc. were opened and transport limits were lifted). Already at the beginning of June 2021, the entire economy was loosened. The spread of the Delta variant in autumn 2021, characterized by higher transmission capacity than the previous dominant Alpha variant, caused the 4th wave of the COVID-19 pandemic in Europe and Poland [11]. In Poland, the peak of the 4th wave occurred in the second half of November.

At the beginning of January 2021, it was announced that a registration for COVID-19 vaccination would start on schedule on 15 January 2021 [12]. The vaccination of Polish medical staff (the so called ‘zero’ group) started on 27 December 2020. Finally, the National Vaccination Program was launched on 25 January 2021 [12]. On 31 May 2021, the total number of vaccinations exceeded 20 million [13]. On 15 June 2021, the number of fully vaccinated people exceeded 10 million [14]. On 4 November 2021, the number of fully vaccinated Polish citizens passed 20 million. On 15 December 2021, the number of people who received the third dose (booster) of the vaccine exceeded 5 million. On 31 December 2021, the number of citizens vaccinated with at least one dose was 56.64 percent of the country’s population [15].

The SARS-CoV-2 pandemic, as a new, unexpected and unpredictable phenomenon, has had a profound impact on all aspects of life in the global population. Estimating the losses and long-term effects of this crisis is and will be the subject of many studies now and in the future. In the scientific literature, many COVID-19-related problems have been widely discussed so far, including also mental health [16,17]. The numerous negative psychological consequences of the pandemic itself and related quarantine were documented [18,19,20].

A high level of stress and anxiety is probably due to the long-lasting state of the epidemic and associated restrictions, and the fact of escalation of fear and suffering. Dalpati et al. indicated the lack of control in the current situation and the uncertainty sustained over time as the important triggers of stress symptoms [21]. In turn, Kerr et al. noted that the symptoms of depression may be derived from the lack of hope and failure to see the end of the pandemic [22].

There is a strong recommendation from the Lancet’s COVID-19 Commission Mental Health Task Force to monitor the mental health of populations over the next few years given the many warning signs that persist, beyond the first years of the SARS-CoV-2 pandemic [23,24].

Therefore, the aim of the presented study was to assess the impact of the COVID-19 pandemic on the mental state of Poles, including the level of self-reported depression, anxiety, stress, and sleep problems based on validated questionnaires. The additional aim was to identify factors associated with mental health outcomes.

## 2. Materials and Methods

### 2.1. Sample Composition

The study sample was calculated with the use of the formula for a finite population [25]. The required group size was set at 1068 on the basis of an estimated number of 20 million adult Polish citizens with access to the Internet [26], an estimated fraction size of 50% (a standard if the fraction is unknown), a significance level of α = 0.05, and a permissible error (e) = of 3%. The study group consisted mainly of employees of medical universities in Poland. Three thousand e-mails were sent to the above Poles with a request to join the study voluntarily. The e-mail contained a link to the research questionnaire and all necessary information about the study’s purpose and rules of participation. Moreover, potential respondents were able to download the link to the study questionnaire from the social platforms Facebook, Twitter, and LinkedIn. A total of 1559 respondents (all who agreed to participate in the study) were taken into consideration. However, based on the inclusion criteria of the study and incomplete questionnaires by 253 healthcare workers, 1306 respondents were finally included in the study. The response rate, defined as the number of adequately completed online forms, was 43.53%. Respondents involved in the study met the following inclusion criteria: age of at least 18 years, current residency in Poland, and informed consent to take part in the study. They were instructed about the voluntary nature of their participation, the study objectives, and the safety and anonymity of the project. Subjects, who did not meet the inclusion criteria, were excluded.

### 2.2. Ethical Approval

The research project received a positive opinion from the local bioethics committee at Poznan University of Medical Sciences (No. 22/21,14.01.2021). The committee confirmed that the study had no features of a medical experiment. All the canons established in the Personal Data Protection Act were followed for data collection. The identity of participants was anonymous, and no personal data of surveyed subjects were registered. Therefore, the procedures followed were in accordance with the Declaration of Helsinki, as revised in 2008.

### 2.3. Study Design

A prospective, cross-sectional web-based survey design was adopted. The study horizon was from 1 June 2021 to 31 December 2021. In order to minimize the risk of SARS-Cov2 infection among the recruited subjects, the survey was performed with the use of the Computer-Assisted Web Interview (CAWI) method. The Google Forms questionnaire was created and disseminated electronically by means of social media. Standardized questionnaires in the Polish language were used as research tools: Depression, Anxiety, and Stress Scale (DASS-21) [27,28,29], and the Insomnia Severity Index (ISI) (Supplementary Materials Files S1–S3) [30,31,32].

The DASS-21 is a 21-item questionnaire that is commonly used as a tool for the assessment of the levels of depression, anxiety, and stress in the population aged 14 years and above. The respondents are asked to report on experiences during the previous week. The DASS-21 consists of three main parts: depression, anxiety, and stress. In each part, there are seven questions, with a Likert scale ranging from zero (0 = Did not apply to me) to three (3 = Applied to me very much or most of the time). To categorize symptoms of stress, anxiety, and depression, the cut-off points discussed by Antony et al. [33] are used: no symptoms, mild, moderate, severe, severe and extremely severe. The DASS-21 has acceptable reliability and good validity [34].

The ISI is a self-reported Likert scale instrument assessing sleep problems and the severity of all-day components of insomnia. This scale includes 7 items that are rated on a 0–4 scale. Total scores range from 0 (best outcome) to 28 (worst outcome) and allow estimation of the severity of insomnia symptoms as follows: absence of insomnia (0–7), subthreshold insomnia (8–14), moderate insomnia (15–21), and severe insomnia (22–28) [31,35].

### 2.4. Data Analysis

Counts and percentages were used to present non measurable variables. For categorical data, the coefficients for a specified level were compared to the reference level. The linear regression model was used to assess the relationship between the analyzed parameters. In the next step, multiple regression analysis was performed in order to determine significant predictors adjusted for the presence of other variables. The results are presented as regression coefficients and its 95% confidence intervals (95%CI). A *t*-student test was used to check if the denoted regression coefficient is significant. A *p* value of less than 0.05 was considered significant. The statistical analysis was performed using the Statistica data analysis software v. 13.3 (TIBCO Software Inc. 2017, Palo Alto, CA, USA; http://statistica.io accessed on 1 March 2022).

## 3. Results

### 3.1. Descriptive Analysis of the Study Population

Of the total study sample (1306 respondents involved in the study), 77.79% (*n* = 1016) were females at a mean age of 34.89 ± (14.79). Almost two thirds (*n* = 813) had higher education, over half of the sample (*n* = 737) had a salary as a source of income, and almost half of the sample (*n* = 608) lived in a city of over 500,000 inhabitants. Two-thirds of the participants lived with a family, and 48.32% (*n* = 631) worked normally during the COVID-19 pandemic. A similar percentage (47.70%, *n* = 623) estimated their health status as very good. Of the total study sample, 46.02% (*n* = 601) reported having comorbidities. In the question related to the regular use of stimulants, 38.54% (*n* = 503) responded that they use alcohol, and 13.95% (*n* = 182) admitted to smoking cigarettes. Of the total study sample, one-fourth (*n* = 601) indicated that they had an infection with SARS-CoV-2. Regarding the vaccination against SARS-CoV-2, 88.97% (*n* = 1162) reported being fully vaccinated against COVID-19 (Table 1).

### 3.2. Percentages of Respondents with Different Symptomatologies

As a result of the study, we found that out of the total sample, 49.62% of people had a normal level of depression, 56.51% of people had a normal level of anxiety, and 38.74% of people had a normal level of stress. Extremely severe level of depression, anxiety, and stress was observed among 5.05%, 5.67%, and 0.92% of respondents, respectively (Table 2).

### 3.3. Analysis of Sleep Disturbances

On the basis of a chi-square test analysis, no clinically significant levels of sleep disturbances were found in over half of the population (55.25%). Only 2.29% of the respondents represented severe level of sleep disturbances (Table 3).

### 3.4. Regression Univariable Analysis for the Psychiatric Symptoms Outcome: Depression, Anxiety, Stress, and Sleep Disturbance among Poles during the COVID-19 Pandemic

Additional univariable regression analysis, which was performed for confounders that might influence the level of psychiatric symptoms outcome (depression, anxiety, stress and sleep disturbance), showed that according to anxiety and stress, females scored higher than males in the analyzed population (Coeff.: −1.09, *p* = 0.012; Coeff.: −2.01, *p* < 0.0001, respectively). The age ranges show statistically significant differences with the symptomatology (depression and anxiety) (Coeff.: −0.04, *p* = 0.010; Coeff.: −0.03, *p* = 0.003, respectively). Similarly, there was a relationship between living alone and developing the symptoms of depression and anxiety. Depending on gender, and age, the only symptomatology showing no statistically significant differences were sleep disturbances. None of them show significant differences with education, place of work during the COVID-19 pandemic, use of alcohol, and use of drugs. The worse people’s self-estimated health status, the more likely they were to experience depression, anxiety, stress, and sleep disturbances. Similarly, the presence of comorbidities and regular use of nicotine significantly increased the risk of any of the analyzed psychiatric symptoms and sleep disorders. Confirmed infection with SARS-CoV-2 virus statistically significantly increased the risk of depression, anxiety, and sleep disturbances (Coeff.: 1.48, *p* = 0.044; Coeff.: 1.62, *p* = 0.004; Coeff.: 1.52, *p* = 0.005). On the contrary, being vaccinated against SARS-CoV-2 statistically significantly decreased the risk of anxiety and stress (Coeff.: −0.91, *p* = 0.027; Coeff.: −1.58, *p* = 0.001) (Table 4). 

### 3.5. Multiple Regression Analysis for the Psychiatric Symptoms Outcome: Depression, Anxiety, Stress, and Sleep Disturbances among Poles during the COVID-19 Pandemic

The performed multiple regression analysis for confounders that may influence the level of psychiatric symptoms outcome (depression, anxiety, stress, and sleep disturbance) showed that the depression level was significantly associated with age (Coeff.: −0.11, *p* < 0.0001), living alone (Coeff.: 1.54, *p* = 0.003), health status (*p* < 0.0001), and the use of nicotine (Coeff.: 1.99, *p* = 0.001). Younger, smoking Poles, living alone, with bad health conditions represented the highest level of depression. Moreover, the level of anxiety was significantly associated with age (Coeff.: −0.08, *p* < 0.0001) and health status (*p* < 0.0001). Younger Poles with poor health conditions represented the highest level of anxiety. The level of stress depended on gender (Coeff.: −1.61, *p* < 0.0001), age (Coeff.: 0.08, *p* < 0.0001), health status (*p* < 0.0001), use of nicotine (Coeff.: 1.59, *p* = 0.009), and being vaccinated against SARS-CoV-2 (Coeff.: −1.18, *p* = 0.006). Younger females using nicotine with bad health conditions represented the highest level of stress. On the contrary, being vaccinated against SARS-CoV-2 statistically significantly decreased the risk of stress. Sleep disturbances depended on age (Coeff.: −0.03, *p* = 0.022), health status (*p* < 0.0001), the presence of comorbidities (Coeff.: 1.73, *p* = 0.030), and regular use of drugs (Coeff.: 2.68, *p* = 0.013). Younger Poles with comorbidities and who were taking drugs were more likely to experience sleep disturbances (Table 5). 

## 4. Discussion

### 4.1. Main Findings

In the present study, the prevalence of depression, anxiety, stress, and sleep disturbances were as follows: 50.38%, 43.49%, 61.26%, and 44.74%, respectively. Poor self-estimated health status, the presence of comorbidities, and regular use of nicotine significantly increased the risk of any of the analyzed psychiatric symptoms and sleep disorders. The depression level was significantly associated with age, living alone, health status, and the use of nicotine. Moreover, the level of anxiety was significantly associated with age and health status. The level of stress depended on gender, age, health status, use of nicotine, and being vaccinated against SARS-CoV-2. Sleep disturbances depended on age, health status, the presence of comorbidities, and regular use of drugs.

### 4.2. The Prevalence of Depression, Anxiety and Stress

Since the beginning of 2020, the predominant psychopathologies in societies suffering from the SARS-CoV-2 pandemic have been anxiety, stress, depression, and insomnia [36]. Before the COVID-19 pandemic, a large-scale epidemiological study reported a one year prevalence of any psychiatric disorder of 10.5% in Poland (23% for the life-time prevalence) [37). In the same study, the prevalence of depression was 3% for GAD (*Generalized Anxiety Disorder 7-item Scale*, GAD-7) 1%, and 1% for PTSD (Post-Traumatic Stress Disorder) [37].

Currently, 350 million people worldwide suffer from depression [38]. It is estimated that every fifth woman and every tenth man has at least one episode of depression during their lifetime. Depression is most often diagnosed in people between 20 and 40 years of age. Women suffer from it twice as often. In Poland, the number of people suffering from depression is already estimated at 1.5 million [38]. The present results from the second half of 2021 could be compared with a study conducted on 382 adults by Chirkowska-Smolak et al., who revealed that every fifth respondent experienced at least moderate symptoms of depression, anxiety, and stress, including those with severe and extremely severe scores obtained by 11.2% of respondents for depression, 10.6% for anxiety, and 9.1% for stress [39]. In another Polish study conducted on 2172 adults, Juchnowicz et al. showed that moderate to extremely severe scores of depression, anxiety, and stress were reported by 43.4%, 27.3%, and 41.0% of the participants, respectively [40]. In turn, Dziedzic et al. investigated the mental health of nurses during the SARS-CoV-2 pandemic and found symptoms of depression in 53.4% of individuals, anxiety in 72.3% of them, and moderate or high levels of stress in 49.5% of them [41]. This can be explained by the fact that the respondents were members of medical staff.

Similar data come from a survey conducted in eight European countries on the population of 609 adults. It was shown that 18% of participants reported moderate depression, 15% reported a moderate level of anxiety, and 14% reported a moderate level of stress [42]. The French study conducted on almost 70,000 respondents showed that during the national quarantine, high levels of perceived stress, depression, and anxiety were present in 24.7%, 16.1%, and 27.5% of participants, respectively [43]. 

In Asia, in the survey completed by 795 Chinese anesthesiologists the prevalence of depression, anxiety, and stress in this sample were 26.5%, 35.5%, and 19.9%, respectively [44]. Additionally, another Chinese survey, which received a total of 52,730 valid responses, revealed that almost 35% of the respondents experienced psychological distress [45].

In Saudi Arabia, Alyami et al. showed that 41% of study participants had symptoms of depression and 38% had mild, moderate, severe, or extremely severe levels of anxiety [46].

### 4.3. Factors Associated with Poor Mental Health Outcomes (Severe Symptoms of Anxiety, Depression, and Stress)

Interesting comparison of mental health of Polish people between the first three waves of the pandemic and the fourth wave was made by Babicki et al. [20]. As in the presented study, they found that factors that increased anxiety and depression were younger age, female, and reduced earning capacity. It can be assumed that the difference in the results of the fourth wave of the pandemic can be explained by the citizen’s adaptation to the situation and the loosening of restrictions [20].

In terms of gender, as in the presented study, many researchers showed that women have suffered the most psychological distress during the COVID-19 pandemic [40,41,43,46,47,48,49,50,51,52,53]. As it was mentioned above, one of the possible reasons could be the fact that women were often employed in sectors in which they work have been more affected [41,54]. What is more, women were under pressure to join work and home duties, also taking care of family members during the COVID-19 pandemic [43,45,47,50]. However, Hyland et al. proved that depression and anxiety symptoms were associated with being male [55].

As far as age is concerned, the majority of studies confirmed that being young was a risk factor for having mental health problems during the COVID-19 pandemic [47,48,53,55]. However, elderly age was also negatively correlated with psychopathological symptoms in some studies [44,56,57]. Qiu et al. proved that individuals between 18 and 30 years of age or above 60 presented the highest CPDI (COVID-19-related stress) scores [45]. 

In Poland, Dziedzic et al. showed no statistically significant differences in the prevalence of mental health problems with regard to age [41]. Benatov et al. indicated that a younger age (20–29 years) of Polish responders predicted coronavirus-related PTSD (post-traumatic stress disorder risk) [53]. It can be assumed, that many aspects of life in the COVID-19 pandemic are responsible for this, including sudden social isolation and fear of the real world.

Unemployment was found to be associated with a higher level of depression and stress in a global population [47]. In two Polish studies, it was shown that a lower educational level was associated with increased odds of mental health problems during the COVID-19 pandemic [40,51]. During analyzing the potential link between mood disorders and a situation in workplace, job insecurity is found to be one of key factors. The negative assessment of the chances of finding a new job in the event of a job loss was an important moderator of this relationship. The seasonally adjusted registered unemployment rate increased to 6.2% in February 2021 [58]. OECD calculations showed 20% of employment was at risk in Poland due to the pandemic [58]. The epidemic situation resulted in limiting the activities of litters of the national economy in its current form. This was manifested, on the one hand, by the liquidation of jobs and, on the other hand, by employers opening up to forms of employment, allowing for social distancing. One such form was remote work. On 30 September 2021, 5% of Polish employees worked remotely [59].

Abdul Latif et al. conducted a study on the population of 350 Malaysian women between October 2020 and April 2021 and revealed that low education level unemployment and loss of income were significantly associated with a higher psychological burden [60]. Bussè et al. also confirmed that long-term higher depression was predicted by a model built on baseline information where having lower education was a significant predictor [50]. In the Irish study, it was shown that depression and anxiety symptoms were associated with loss of income due to COVID-19 and COVID-19 infection [55]. On the contrary, education level, employment status, and income had no significant association with DASS-21 subscores in the study conducted during the COVID-19 pandemic in Saudi Arabia [48].

Qiu et al. showed that participants’ high score of psychopathological symptoms was associated with higher education, probably because of high self-awareness of their health [45]. Similar findings come from the survey conducted in eight Asian countries with 4479 respondents [18]. Wang et al. reported that respondents with higher education were associated with higher stress, anxiety and depression [18]. The same Chinese author together with Polish researchers compared populations from China and Poland [61]. Polish respondents had significantly higher levels of anxiety, depression, and stress than Chinese. Among others, unemployment and retirement were risk factors for symptoms of anxiety, depression, and stress only for Polish respondents [61].

There was a relationship between living alone and developing the symptoms of depression and anxiety. However, in the study questionnaire, there was no question directly about marital status. In many studies, marital status was negatively correlated with psychopathological symptoms [44]. It was shown that being single, divorced, or widowed marital status was predictive of mental health problems [18,40,47,53]. However, marital status had no significant association with DASS-21 subscores in the study conducted during COVID-19 pandemic in Saudi Arabia [48].

### 4.4. The Prevalence of Insomnia and Sleep Disturbances

In the population of 999 American adults, Dzierzewski et al. [62] found that moderate-to-severe insomnia symptoms prevalence was even twice as high (25.5%), which is lower compared to frontline health care workers during the COVID-19 pandemic [56], but consistent with general samples [16].

In a study conducted on 476 American adults, it was shown that the mean score for ISI was 6.9 (5.2), with 11.5% of subjects having clinical insomnia (defined as ISI ≥ 15), of whom 9% had severe clinical insomnia [63]. In turn, a Chinese study conducted on university students showed that anxiety and depression were positively correlated with insomnia [64]. Duan et al. showed that the prevalence of insomnia was 32.73%, of anxiety 15.43%, and of depression 62.91%. Female students were more likely to have insomnia. Insomnia severity was positively correlated to anxiety severity, and anxiety severity correlated positively to depression severity [64]. Alyami et al. showed that there was a strong association between sleep disturbances and psychological distress [46].

### 4.5. Positive History of Health Problems

In the presented study, the worse people’s self-estimated health status, the more likely they were to experience depression, anxiety, stress, and sleep disturbances. Similarly, the presence of comorbidities significantly increased the risk of any of the analyzed psychiatric symptoms and sleep disorders. In the literature it was reported that people who had a diagnosis of psychiatric illness in the pre-pandemic time, were significantly more likely to have higher mean DASS depression, anxiety, and stress subscale scores and ISI scores [18,51]. It was also indicated that patients with chronic illnesses, diagnosed in pre-pandemic time, were at greater risk of fear, anxiety, and depressive symptoms due to unexpected difficulties in the functioning of the health care system [65].

### 4.6. Use of Nicotine, Alcohol and Drugs

It was shown that regular use of nicotine significantly increased the risk of any of the analyzed psychiatric symptoms and sleep disorders. In the presented, study participants who admitted to taking drugs were more likely to experience sleep disturbances. The presence of anxiety, stress, and other mental problems related to the COVID-19 pandemic, increased the risk of involvement with or worsens the use of alcohol, nicotine, and other addictive substances as a maladaptive coping strategy [66]. On the base of 45 cross-sectional studies conducted in 2020, alcohol use was on the rise overall worldwide, as was the use of other addictive substances [67]. It was shown that people who experienced more severe symptoms of depression and stress declared increased alcohol use during the COVID-19 pandemic [68]. In the literature, it is underlined that ‘substance use appears to have an autonomous dynamism in relation to the pandemic and the consequent psychopathology, being in a “loose” causal relationship with it’ [66].

### 4.7. Status of Vaccination against COVID-19 and Confirmed Infection with SARS-CoV-2 Virus in the Past

Confirmed infection with the SARS-CoV-2 virus statistically significantly increased the risk of depression, anxiety, and sleep disturbances, while being vaccinated against SARS-CoV-2 statistically significantly decreased the risk of anxiety and stress. It is in line with the results obtained by Huang et al., who observed that at 6 months after the COVID-19 pandemic began, patients were at greater risk of sleep difficulties, and anxiety or depression [69]. In the literature it is also reported that mental health problems might be one of the reasons for patient’s lack of vaccine acceptance [70]. However, it is associated mainly with lack of trust in science, governments, and so-called ‘conspiratorial thinking’ about COVID-19 [71].

## 5. Study Limitations and Strengths

Some limitations should be considered in the interpretation of the obtained results. The first limitation is that although the number of participants was large, it represents only a small percentage of the Polish population, so caution is therefore necessary before generalizing the study findings. It should be underlined that the mentioned problem is also found in all large epidemiologic projects and does not systematically mean that the self-selection bias changed the results. Low response rates in epidemiological studies have been shown to have a marginal impact on prevalence and association measures [72,73]. Another important limitation is the fact that the sample structure does not reflect the gender structure in the general population of Polish nationality. More women (78%) took part in the survey, which results from the more significant activity of women in social media. Moreover, the questionnaire used in the presented study to identify psychopathological symptoms remains only a screening tool, but not a diagnostic one, although it has been validated. However, a high score on these validated tests is highly correlated with the presence of mental health problems.

Third, it should be pointed out that it is not possible to establish whether the pandemic itself directly caused psychopathological symptoms.

However, it should be emphasized that, to our knowledge, this is one of the few studies conducted during the SARS-CoV-2 pandemic in Poland and it undeniably brings a new look at the mental health of Polish citizens in the analyzed period. Therefore, it highlights the importance of addressing the mental state of the Poles and how sociodemographic and pandemic-related variables may influence this psychosymptomatology.

## 6. Conclusions

In conclusion, this study highlights the importance of addressing the mental health of Polish citizens, and more research and solutions are needed to investigate the scale of psychopathological disorders during the COVID-19 and post-COVID-19 era. As far as mental health professionals are concerned, interventions for the treatment of mental disorders resulting from the pandemic should be put in place, and special attention should be paid to the most vulnerable groups, including, among others, younger women, people living alone, and those having comorbidities, bad self-estimated health status, and smoking cigarettes, with confirmed infection with the SARS-CoV-2 virus in the past. It should be underlined that being vaccinated against SARS-CoV-2 statistically significantly decreased the risk of anxiety and stress.

Our findings confirmed the necessity to monitor psychological adaption over time in general and at-risk individuals. What is more, there is a strong need to make information widely available to the public on how to overcome mental health problems and where to find professional help. Societies should be educated about the role of personal, supportive relationships in daily life [74].

## Figures and Tables

**Table 1:** General characteristics of patients (n = 1306).

**Group size**	**Total**	1306
	Female n (%)	1016 (77.79)
	Male n (%)	290 (22.21)
**Age**	Total Mean ± (SD)	34.89 ± (14.79)
**Education**	Higher n (%)	813 (62.25)
	Secondary n (%)	461 (35.30)
	Vocational n (%)	25 (1.91)
	Primary n (%)	7 (0.54)
**Source of income**	Salary n (%)	737 (56.43)
	Pension n (%)	60 (4.59)
	Supported by parents n (%)	495 (37.90)
	Unemployed n (%)	14 (1.07)
**Place of residence**	Village n (%)	276 (21.13)
	Town of <10,000 inhabitants n (%)	199 (15.24)
	Town of 10,000–100,000 inhabitants n (%)	125 (9.57)
	City of 100,000–500,000 inhabitants n (%)	98 (7.50)
	City of over 500,000 inhabitants n (%)	608 (46.55)
**Running the household alone or with the family**	Alone n (%)	301 (23.05)
	With family n (%)	1005 (76.95)
**Place of work during the COVID-19 pandemic**	Hybrid n (%)	211 (28.63)
	in the workplace n (%)	377 (51.15)
	remotely at home n (%)	149 (20.22)
**Health status**	Excellent n (%)	144 (11.03)
	Very good n (%)	623 (47.70)
	Good n (%)	455 (34.84)
	not very good n (%)	75 (5.74)
	Bad n (%)	9 (0.69)
**The presence of comorbidities**	n (%)	601 (46.02)
**Regular use of stimulants**	Alcohol n (%)	503 (38.54)
	Nicotine n (%)	182 (13.95)
	Drugs n (%)	27 (2.07)
**Confirmed infection with SARS-CoV-2 virus**	n (%)	340 (26.03%)
**Being vaccinated against the virus SARS-CoV-2**	n (%)	1162 (88.97%)

**Table 2:** Evaluation of the psychiatric symptoms: depression, anxiety, and stress among Poles (n = 1306).

Variables	General Mean ± SD	Normal %	Mild %	Moderate %	Severe %	Extremely Severe %
Level of depression	10.71 ± 8.46	49.62	16.77	20.90	7.66	5.05
Level of anxiety	7.25 ± 6.53	56.51	9.80	21.59	6.43	5.67
Level of stress	13.38 ± 7.46	38.74	39.51	17.23	3.60	0.92

**Table 3:** Evaluation of the sleep disturbances among Poles n = 1306.

Variables	General Mean ± SD	No Clinically Significant %	Subthreshold %	Moderate Severity %	Severe %
**Level of sleep disturbances**	7.80 ± 5.69	55.26	31.86	10.59	2.29

**Table 4:** Regression univariable analysis for the psychiatric symptoms outcome: depression, anxiety, and stress and sleep disturbance among Poles during the COVID-19 pandemic.

	Level of Depression		Level of Anxiety		Level of Stress		Sleep Disturbance	
Variable	Coeff. (95% CI)	*p*-Value	Coeff. (95% CI)	*p*-Value	Coeff. (95% CI)	*p*-Value	Coeff. (95% CI)	*p*-Value
**Gender:**								
Female ref.								
Male	−0.69 [−1.80, 0.41]	0.215	−1.09 [−1.94, −0.24]	0.012	−2.01 [−2.98, −1.04]	<0.0001	−0.48 [−1.25, 0.28]	0.219
**Age**	−0.04 [−0.07, −0.001]	0.01	−0.03 [−0.06, −0.012]	0.003	−0.19 [−0.04, 0.007]	0.163	0.02 [−0.01, 0.04]	0.056
**Education:**								
Primary ref.								
Vocational	4.51 [−2.68, 11.72]	0.219	1.85 [−3.68, 7.39]	0.511	1.88 [−4.47, 8.23]	0.561	2.48 [−2.36, 7.33]	0.315
Secondary	2.58 [−3.73, 8.90]	0.422	3.04 [−1.81, 7.90]	0.219	2.89 [−2.68, 8.46]	0.309	1.59 [−2.66, 5.85]	0.462
Higher	1.83 [−4.46, 8.13]	0.568	1.59 [−3.25, 6.43]	0.519	2.80 [−2.75, 8.36]	0.323	1.44 [−2.79, 5.69]	0.504
**Place of work during COVID-19 pandemic:** Unemployed ref.								
Hybrid	−0.06 [−1.79, 1.67]	0.207	−0.68 [−1.96, 1.59]	0.3	0.91 [−0.54, 2.36]	0.221	−0.25 [−1.42, 0.91]	0.666
in the workplace	0.07 [−1.57, 1.72]	0.933	0.32, [−0.94, 1.59]	0.616	0.51 [−1.24, 2.25]	0.571	0.61 [−0.55, 1.77]	0.305
remotely at home	−0.06 [−1.79, 1.67]	0.945	−0.30 [−1.64, 1.03]	0.655	0.53 [−0.99, 2.06]	0.492	−0.25 [−1.46, 0.94]	0.672
**Whether you live alone or with your family:**								
With family ref.								
Alone	2.08 [0.99, 3.16]	<0.0001	1.00 [0.16, 1.84]	0.019	0.53 [−0.42, 1.50]	0.272	0.07 [−0.71, 0.85]	0.857
**Health status:**								
Excellent ref.								
Very good	3.71 [2.29, 5.13]	<0.0001	2.06 [0.95, 3.17]	<0.0001	3.66 [2.41, 4.92]	<0.0001	1.62 [0.58, 2.67]	0.002
Good	7.19 [5.72, 8.66]	<0.0001	4.60 [3.45, 5.75]	<0.0001	6.98 [5.68, 8.28]	<0.0001	4.45 [3.38, 5.53]	<0.0001
not very good	12.89 [10.71,15.09]	<0.0001	9.18 [7.47, 10.89]	<0.0001	11.11 [9.18, 13.04]	< 0.0001	6.95 [5.38, 8.51]	<0.0001
bad	19.33 [14.04, 24.62]	<0.0001	13.51 [9.38, 17.64]	<0.0001	15.31 [10.65, 19.98]	<0.0001	10.38 [6.76, 14.00]	<0.0001
**The presence of comorbidities**	2.13 [1.22, 9.72]	<0.0001	1.78 [1.08, 6.43]	<0.0001	1.99 [1.18, 12.46]	<0.0001	1.88 [1.24, 6.91]	<0.0001
**Use of alcohol**	0.04 [−0.90, 0.98]	0.932	0.24 [−0.48, 0.97]	0.506	0.51 [−0.32, 1.34]	0.231	0.31 [−0.35, 0.96]	0.368
**Use of nicotine**	2.57 [1.25, 3.89]	<0.0001	2.34 [1.32, 3.35]	<0.0001	2.38 [1.22, 3.55]	<0.001	1.26 [0.30, 2.21]	0.01
**Use of drugs**	1.09 [−2.13, 4.32]	0.507	0.39 [−2.10, 2.88]	0.759	−0.73 [−3.58, 2.11]	0.613	1.79 [−0.46, 4.05]	0.119
**Confirmed infection with SARS-CoV-2 virus**	1.48 [0.037, 2.92]	0.044	1.62 [0.51, 2.73]	0.004	1.24 [−0,03, 2.51]	0.057	1.52 [0.45, 2.58]	0.005
**Vaccination against the virus SARS-CoV-2**	−0.97 [−2.02, 0.08]	0.071	−0.91 [−1.73, −0.11]	0.027	−1.58 [−2.51, −0.65]	0.001	−0.47 [−1.21, 0.26]	0.208

*p*-value based on the *t*-student test showing the significance of regression model coefficient.

**Table 5:** Multiple regression analysis for the psychiatric symptoms outcome: depression, anxiety, stress, and sleep disturbances among Poles during the COVID-19 pandemic.

	Level of Depression		Level of Anxiety		Level of Stress		Sleep Disturbances	
Variable	Coeff. (95% CI)	*p*-Value	Coeff. (95% CI)	*p*-Value	Coeff. (95% CI)	*p*-Value	Coeff. (95% CI)	*p*-Value
**Gender:**								
Female ref.								
Male	-	-	-	-	−1.61 [−2.51, −0.70]	<0.0001	-	-
**Age**	−0.11 [−0.13, −0.07]	<0.0001	−0.08 [−0.11, −0.06]	<0.0001	−0.08 [−0.11, 0.05]	<0.0001	−0.03 [−0.05, 0.003]	0.022
**Whether you live alone or with your family:**								
With family ref.								
Alone								
	1.54 [0.54, 2.54]	0.003	-	-	-	-	-	-
**Health status:**								
Excellent ref.								
Very good	4.01 [2.62, 5.39]	<0.0001	2.17 [1.08, 3.26]	<0.0001	3.65 [2.41, 4.88]	<0.0001	1.61 [0.57, 2.66]	0.002
Good	8.26 [6.81, 9.72]	<0.0001	5.14 [3.97, 6.30]	<0.0001	7.45 [6.15, 8.75]	<0.0001	4.46 [3.34, 5.58]	<0.0001
not very good	14.35 [12.18, 16.53]	<0.0001	9.70 [7.96, 11.44]	<0.0001	11.61 [9.67, 13.55]	<0.0001	6.96 [5.32, 8.59]	<0.0001
bad	21.62 [16.42, 26.81]	<0.0001	14.99 [10.93, 19.05]	<0.0001	17.06 [12.47, 21.65]	<0.0001	10.58 [6.92, 14.23]	<0.0001
**The presence of comorbidities**	-	-	-	-	-	-	1.73 [1.07, 8.93]	0.03
**Use of nicotine**	1.99 [0.79, 3.20]	0.001	-	-	1.59 [0.41, 2.77]	0.009	-	-
**Use of drugs**	-	-	-	-	-	-	2.68 [0.56, 6.59]	0.013
**Vaccination against the virus SARS-CoV-2**	-	-	-	-	−1.18 [−2.04, −0.33]	0.006	-	-

*p*-value based on the *t*-student test showing the significance of regression model coefficient.

## Data Availability

The data that support the findings of this study are available from the corresponding author (K.H., karhof@tlen.pl or karolinahoffmann@ump.edu.pl), upon reasonable request.

## Supplementary Materials

The following supporting information can be downloaded at: https://www.mdpi.com/article/10.3390/ijerph20032000/s1, File S1: The Mental Health of Poles during the COVID-19 Pandemic; File S2: DASS-21- Depression Anxiety Stress Scale Test; File S3: Insomnia severity index.
