# Supplementary material for: The Mental Health of Poles during the COVID-19 Pandemic
Source: Int J Environ Res Public Health. 2023 Jan 21;20(3):2000. doi: 10.3390/ijerph20032000 (PMC9914975; doi:10.3390/ijerph20032000)
Supplement: Supplementary file 1 [file ijerph-20-02000-s001.zip › ijerph-2161386-supplementary.pdf]

Supplementary Material File S1

The Mental Health of Poles during the COVID-19 Pandemic

We encourage everyone over the age of 18 to complete this questionnaire: The Mental Health of Poles during the COVID-19 Pandemic

Dear Sirs,

Department of Internal Diseases, Metabolic Disorders and Arterial Hypertension as well as the Pharmacoeconomics and Social Pharmacy Centre of the Poznan University of Medical Sciences are surveying the public opinion pertaining to the impact of Covid-19 pandemic on mental health of Poles.

The survey aims to collect information necessary for a scientific analysis and to prepare a strategy for dealing with health threatening situations.

The survey is safe, free of charge and anonymous. Personal data of respondents shall not be recorded. Data which could be used at a later date to identify the survey participants shall not be used.

In agreeing to take part in the survey you will be asked to respond to questions in an on-line survey which we will use to collect the required demographic information, assess COVID-19 contact history, COVID-19 knowledge and preventative means as well as availability of health related information with regard to COVID-19. We will also assess the impact of the pandemic on your health related behaviours.

Every respondent may approach the authors for information regarding the survey questions and to obtain a report once the survey has ran its course.

Consent to take part in the survey

I have read and understood the information pertaining to the aforementioned survey. By responding to this questionnaire I voluntarily consent to participating in the survey and I am aware that at any time I can withdraw my consent to participate in further parts of the survey without providing a reason.

Pursuant to the Polish law (Personal Data Protection Act of 10 May 2018), this survey shall be anonymous .

1. **Gender: ***
- female
- male

2. **Age: ***
- 18-24
- 25-40
- 41-60
- more than 60.

3. **Education: ***
- primary
- middle school
- basic vocational
- high school
- university

4. **Your place of residence: ***
- village
- town, up to 50 thousand residents
- town, up to 100 thousand residents
- city, up to 250 thousand residents
- city, more than 250 thousand residents.

5. **What is your professional activity status? ***
- Pupil/student
- Professionally active
- Unemployed
- Pensioner
- On benefits.

6. **If you are professionally active, what type of work do you do? ***
- Blue-collar
- White-collar

7. **If you are professionally active, where do you work during the pandemic? ***
- At a workplace
- I work from home
- Currently I am not working as my workplace has closed down / I have closed down my business
- Currently I am not working as my workplace has closed down / I have closed down my business, and I am afraid of losing my job
- I have just lost my job / closed my business because of the market situation brought on by the pandemic.

8. **If you are professionally active, then is there an increased COVID-19 infection risk where you work? ***
    - Yes, the risk is very high
- Yes, the risk is high
- Yes, the risk is moderate
- Yes, the risk is low

- There is no such risk

9. How would you describe your current state of health? *

- Excellent
- Very good
- Good
- Not so good
- Poor

10. Which chronic illness have you been diagnosed with (select as many as required) *

  - Obesity
  - High blood pressure
  - Angina Pectoris (coronary heart disease)
  - Prior heart attack
  - Prior stroke
  - Diabetes
  - Chronic obstructive pulmonary disease (COPD)
  - Osteoarthritis
  - Spondyloarthritis
  - Rheumatoid arthritis
  - Airborne or food allergy
  - Cancer
  - Asthma
  - Depression
  - I have not been diagnosed with any diseases

11. Current body weight (kg):

……………………………………………………………

12. Height (cm): *

…………………………………………………………….

13. Did you suffer from any of the following symptoms during the coronavirus (COVID-19) pandemic? Tick the relevant ones (you may select more than one answer) *

- Persistent fever (above 38°C for at least 1 day)
- Shivers
- Headache
- Muscle pain
- Cough
- Difficulties with breathing
- Dizziness
- Runny nose
- Sore throat
- Sudden loss of smell or taste
- I did not have any of the above symptoms

14. Were you in direct contact with a person infected with the coronavirus (Covid-19)? *

- Yes
- No

15. Were you in contact with a person with a suspected coronavirus (Covid-19) infection or with pathogens? *

- Yes
- No

16. Were you on compulsory quarantine? *

- no
- yes, once
- yes, twice
- yes, more than twice

17. Did you have a coronavirus test done? *

- yes
- no
-

18. Your test result was: *

- positive
- negative

19. Were you hospitalised at any time during the pandemic because of the COVID-19 coronavirus infection? *

- Yes
- No

20. If you were hospitalized due to a COVID-19 infection, was it necessary to resort to mechanical ventilation (ventilator)? *

- Yes
- No

21. Did you suffer from any complications due to the COVID-19 coronavirus infection? *

- Yes
- No

22. **Do you use stimulants?**

a) No,
b) Yes, nicotine
c) Yes, alcohol
d) Yes, drugs

***** the question is mandatory

Supplementary Material File S2. DASS-21- Depression Anxiety Stress Scale Test

Please read each statement and circle a number 0, 1, 2 or 3 which indicates how much the statement applied to you over the past week. There are no right or wrong answers. Do not spend too much time on any statement.

The rating scale is as follows:

0 Did not apply to me at all

1 Applied to me to some degree, or some of the time

2 Applied to me to a considerable degree or a good part of time

3 Applied to me very much or most of the time

1 (s)	I found it hard to wind down	0	1	2	3
2 (a)	I was aware of dryness of my mouth	0	1	2	3
3 (d)	I couldn't seem to experience any positive feeling at all	0	1	2	3
4 (a)	I experienced breathing difficulty (e.g. excessively rapid breathing, breathlessness in the absence of physical exertion)	0	1	2	3
5 (d)	I found it difficult to work up the initiative to do things	0	1	2	3
6 (s)	I tended to over-react to situations	0	1	2	3
7 (a)	I experienced trembling (e.g. in the hands)	0	1	2	3
8 (s)	I felt that I was using a lot of nervous energy	0	1	2	3
9 (a)	I was worried about situations in which I might panic and make a fool of myself	0	1	2	3
10 (d)	I felt that I had nothing to look forward to	0	1	2	3
11 (s)	I found myself getting agitated	0	1	2	3
12 (s)	I found it difficult to relax	0	1	2	3
13 (d)	I felt down-hearted and blue	0	1	2	3
14 (s)	I was intolerant of anything that kept me from getting on with what I was doing	0	1	2	3
15 (a)	I felt I was close to panic	0	1	2	3
16 (d)	I was unable to become enthusiastic about anything	0	1	2	3
17 (d)	I felt I wasn't worth much as a person	0	1	2	3
18 (s)	I felt that I was rather touchy	0	1	2	3
19 (a)	I was aware of the action of my heart in the absence of physical exertion (e.g. sense of heart rate increase, heart missing a beat)	0	1	2	3
20 (a)	I felt scared without any good reason	0	1	2	3
21 (d)	I felt that life was meaningless	0	1	2	3

Supplementary Material File S3.

Insomnia Severity Index

The Insomnia Severity Index has seven questions. The seven answers are added up to get a total score. When you have your total score, look at the 'Guidelines for Scoring/Interpretation' below to see where your sleep difficulty fits.

For each question, please CIRCLE the number that best describes your answer.

Please rate the CURRENT (i.e. LAST 2 WEEKS) SEVERITY of your insomnia problem(s).

Insomnia Problem	None	Mild	Moderate	Severe	Very Severe
1. Difficulty falling asleep	0	1	2	3	4
2. Difficulty staying asleep	0	1	2	3	4
3. Problems waking up too early	0	1	2	3	4

4. How SATISFIED/DISSATISFIED are you with your CURRENT sleep pattern?

Very Satisfied	Satisfied	Moderately Satisfied	Dissatisfied	Very Dissatisfied
0	1	2	3	4

5. How NOTICEABLE to others do you think your sleep problem is in terms of impairing the quality of your life?

Not at all Noticeable	A Little	Somewhat	Much	Very Much Noticeable
0	1	2	3	4

6. How WORRIED/DISTRESSED are you about your current sleep problem?

Not at all Worried	A Little	Somewhat	Much	Very Much Worried
0	1	2	3	4

7. To what extent do you consider your sleep problem to INTERFERE with your daily functioning (e.g. daytime fatigue, mood, ability to function at work/daily chores, concentration, memory, mood, etc.) CURRENTLY?

Not at all Interfering	A Little	Somewhat	Much	Very Much Interfering
0	1	2	3	4
